# The Ubiquitous Issue of Cross-Mass Transfer: Applications to Single-Use Systems

**DOI:** 10.3390/molecules24193467

**Published:** 2019-09-24

**Authors:** Phuong-Mai Nguyen, Samuel Dorey, Olivier Vitrac

**Affiliations:** 1 LNE, 29 Avenue Roger Hennequin, 78197 Trappes CEDEX, France; phuong-mai.nguyen@lne.fr; 2 Sartorius Stedim FMT S.A.S., avenue de Jouques, CS91051, ZI des Paluds, 13781 Aubagne CEDEX, France; 3 Ingénierie Procédés Aliments, AgroParisTech, INRA, Université Paris-Saclay, 91300 Massy, France

**Keywords:** migration, non-intentionally added substances (NIAS), cross-mass transfer, modeling, risk assessment, single-use, extractables, laminates

## Abstract

The leaching of chemicals by materials has been integrated into risk management procedures of many sectors where hygiene and safety are important, including food, medical, pharmaceutical, and biotechnological applications. The approaches focus on direct contact and do not usually address the risk of cross-mass transfer of chemicals from one item or object to another and finally to the contacting phase (e.g., culture medium, biological fluids). Overpackaging systems, as well as secondary or ternary containers, are potentially large reservoirs of non-intentionally added substances (NIAS), which can affect the final risk of contamination. This study provides a comprehensive description of the cross-mass transfer phenomena for single-use bags along the chain of value and the methodology to evaluate them numerically on laminated and assembled systems. The methodology is validated on the risk of migration i) of ϵ-caprolactam originating from the polyamide 6 internal layer of the overpackaging and ii) of nine surrogate migrants with various volatilities and polarities. The effects of imperfect contacts between items and of an air gap between them are particularly discussed and interpreted as a cutoff distance depending on the considered substance. A probabilistic description is suggested to define conservative safety-margins required to manage cross-contamination and NIAS in routine.

## 1. Introduction

Plastic materials have been identified as a major source of chemicals in food and tap water [1,2,3,4]. In the literature, the mechanism of mass transfer of non-covalently bonded substances originating from materials into the contacting phase is coined “migration”. It is an umbrella term for mass transfer phenomena such as mass diffusion, desorption, reabsorption, partitioning, and volatilization. In pharmaceutical and biotechnical applications, plastic materials are used both as containers and also as packaging materials to keep sterility during and after gamma irradiation [5]. Migrants from sterilized plastic materials are not fundamentally different from their original counterparts and include monomers and oligomers, additives, degradation products, impurities, etc. Plastic materials are strictly regulated for food contact applications in the US (21 CFR 175–179), EU (EU regulation 10/2011/EC), and China (GB 9685-2016) and should comply to strict requirements such as a positive list of authorized substances, maximum acceptable concentration in food, maximum amounts in initial materials, impurities, and good manufacturing practices. By contrast with food contact applications, no reference list of substances has been setup for pharmaceutical applications. Though it is a legal requirement to demonstrate the suitability of a contact material for its intended use [6], no standard or guidance exists to evaluate migration either by experimental tests or by calculations. Migration from production systems such as single-use plastic containers can alter the safety, efficacy, potency, or purity of final drug products [7,8]. Emerging cross-contamination phenomena complicates the management of the migration risks along the supply chain. The repeated crises involving the contamination of foodstuffs by printing ink constituents [9,10,11] and by mineral oils [12,13,14,15] offer good examples of delayed contaminations across one or several materials [16,17]. In the literature, the migration of non-intentionally added substances (NIAS) has been the standard terminology for contaminations occurring without prior identification of the main sources, mechanisms, and pathways. The detection of chemicals of concern in biopharmaceutical applications was mainly retrospective [18,19], whereas current approaches promote prevention [20,21].

Additionally, incorrect descriptions of mass transfer can lead to biased reasoning and underestimate the real level of migration. As an example, the accumulation of apolar substances in aqueous contacting phases is likely to be underestimated either because solubility in water and back-diffusion are misrepresented [22,23,24] or because reactivities in water have been neglected [25,26,27,28,29,30]. Migration modeling reviewed in [31] has been used for decades for authorizing new substances, validating polymer recycling processes, and compliance testing in both the US and EU. It offers a robust methodology to mitigate the risk of contamination regardless of the considered materials, substances, and routes of accretion. Despite calls for unified approaches [32,33,34], migration modeling is comparatively less developed for medical applications [35,36,37]. The needs have been revived in recent years, with the extending concern associated with endocrine-disrupting chemicals [38,39,40,41] and low-dose effects [42]. Very robust methods, derived from food safety concepts, have been proposed to design safe materials and substances [43,44,45,46] under uncertainty. The adaptation of Failure Mode Effects and Criticality Analysis, or FMECA, methods to mass transfer problems is the most promising, as it can cover scenarios with arbitrary complexity, including the migration of one material or one component from another one at an early stage of the supply chain [44]. Safe-by-design concepts have been recently integrated into more global strategies of rapid prototyping and ecodesign of food packaging [47].

This study aims at demonstrating the universal and ubiquitous character of cross-mass transfer between materials and its persistence even when the source of NIAS has been removed. Without a loss of generality, the driving forces, the time-scales, and the extent of cross-mass transfer are evidenced with or without direct contact and discussed via a comprehensive case-study where chemicals are brought by packaging systems commonly used to protect single-use bags devoted to biopharmaceutical applications. It is shown that phenomena are qualitatively and quantitatively foreseeable in time and space, so that the risk of cross-mass transfer can be mitigated with proper preventive approaches. Section 2 introduces a dimensionless formulation of cross-mass transfer, enabling to reveal the common structure of cross-mass transfer kinetics and the practical mathematical relationships to analyze them. The experimental conditions associated with our studied conditions are detailed in Section 3. The main results and their interpretations are analyzed in the Section 4, including the probabilization of the risk of cross-mass transfer. Finally, the findings and recommendations are summarized in the last section.

## 2. Theory of Cross-Mass Transfer 

### 2.1. Scope

It is usually thought that only materials in contact with the bioproducts or materials in its vicinity can release substances [48,49]. Cross-mass transfer between materials is usually included in the risk management procedures through the evasive concept of non-intentionally added substances (NIAS). This theoretical section illustrates essential features of cross-mass transfer on simple examples, which have relevance in single-use applications. Firstly and as reviewed in [50], all non-covalently bounded substances are exchanged without delay between components, whenever they are parts of an assembly, just in contact or placed in a neighborhood. Cross-mass transfer without contact has been firstly described in ultra-high vacuum applications [51], particle accelerators [52], and spacecrafts [53]. The phenomenon is coined outgassing and covers the spontaneous desorption of all substances, whether they are organic or light gases (water, oxygen, hydrogen). NASA [54] and ESA [55] provide extensive lists of outgassing data categorized by materials and type of materials (polymer family, thermosets). Similar phenomena occur at atmospheric pressure for a broad range of aliphatic or aromatic compounds [17]. Though there is a strong effect of distance for non-volatile substances, no direct or permanent contact is needed to trigger the cross-mass transfer of packaging constituents, as shown experimentally with radiolabeled molecules [27,56]. Secondly, there is no memory of previous migrations, and substances are redistributed or leached as soon as a new material, or medium, is introduced or whenever the temperature of the storage is changed. Specific arrangements of materials during storage can accelerate migration either by symmetrizing contacts (same materials in contact, reducing loss with surroundings) or by introducing some periodicity in contacts facilitating inner mass transfer (e.g., outside and inner sides come in contact). The different configurations met in food packaging applications, and the principles to evaluate them, have been extensively reviewed in [31]. 

Quoting van Kampen [57], “no universal form of the diffusion equation exists” to describe the spread of non-covalently bounded substances (migrants) in inhomogeneous media or when several materials are involved. The complications arise when advection (gas or liquid flows) or cycles of volatilization-reabsorption occur. We proposed previously [44] a simplified one-dimensional description of the mass transfer problem for food packaging and related applications, which also covers single-use applications. The entire system, including the fluid medium, is represented as a collection of *m* + 1 layers of thickness ${\left\{l_{j}\right\}}_{j=1\ldots m}$ that are larger than the mean free path of molecules. 

One or several layers may be a gas layer. The coding is sufficiently general to cover all practical cases. The detailed case-studies correspond to two mono-material layers (denoted A and B) in direct contact (m=2) or separated by a gas layer (m=3) and finally put in contact with a liquid medium (F). The order of layers and their presence can evolve along the supply chain. The fluid layer F is indexed *j* = 0, the contact layer *j* = 1, and the most opposite layer is associated with *j* = *m*. The formulation is standardized by replacing the layer-related properties by dimensionless ratios relative to a reference layer (for example, *j* = 1).

### 2.2. Two Case-Studies Relevant for Single-Use Applications

Assessing the risk of leaching of substances aims at estimating the amount of transferred substances (s_A_ and s_B_) to the final bioproduct F in conservative conditions of storage and use. Substances s_A_ and s_B_ are assumed to be additives or residues (monomers, oligomers, catalysts, solvents, polymerization aids), which are endogenous to A and B, respectively. The two case-studies differ from the permanence of contacts between A and B. The case-studies are designed for biotechnological applications, but their analogy with food applications is also given.

In the first case-study, denoted S1, A and B are laminated together within a bilayer film (coded AB) forming the walls of the single bag (or bilayer packaging for food applications) and intended to be filled with F.

In the second case-study, denoted S2, A is the overpackaging (or a secondary packaging for food applications) of the single-use bag B (or a primary packaging for food applications). The contacts between A and B are imperfect and not permanent; material A is separated from B by an air layer, with an assembly coded A-air-B, with A intended to be discarded before filling B with F.

In both cases, the systems AB are stored in stacks (periodic arrangement ABBA|ABBA) for extended periods (step 1) before being put into a contact period with F (step 2). Corresponding cross-mass transfer scenarios are coded in Table 1. Without a lack of generality, the mass transfer descriptions assume that the migrants are present at low concentrations.

### 2.3. Mathematical Models and Their Solutions

#### 2.3.1. Principles

Cross-contamination models are by nature closely related to those used for migration modeling (see early formulations in [25,58] and the recent review in [31]). For simple configurations, migration models are rooted to the exact solutions of self-similar diffusion or conduction problems collected in the reference textbooks of Crank [59], Carslaw and Jaeger [60], and Luikov [61]. Each problem is associated with a dimensionless version of transport equations and initial and boundary conditions. In the context of materials, analytical solutions have been tabulated for systems, including up to two compartments [62]. For more composite systems, finite volume, sorption/desorption isotherms, external mass transfer resistances, and flows complicate the existence of such solutions dramatically, as discussed in [63,64,65,66]. In this section, we keep the engineering practice to replace the real problems with similitude models involving a reduced number of independent and dimensionless variables. Besides being minimalist, dimensionless quantities (Fourier and mass Biot numbers, ratios of characteristics lengths, concentrations, etc.) and their relationships in diffusion equations accept a probabilistic interpretation [67]. In the presence of large discontinuities in transport and thermodynamic properties, the accurate estimation of low contaminations requires specific numerical techniques. We used in this study a finite-volume technique integrating piece-wise exact analytical solutions over small domains [44]. The worst-case migration values at equilibrium are given analytically and can be used for validating the absence of truncation errors.

#### 2.3.2. Common Assumptions

Migration modeling assumes that all migrants are transferable and distributed homogeneously after equilibration (no blooming, no specific crystallization). Additionally, no migrant remains trapped in crystallites or is present above its saturation concentration. The reversibility of sorption/desorption isotherms is used in this study to setup experiments with controlled initial distributions, without affecting the generality of the presented conclusions.

#### 2.3.3. Transport Equations

We assume that the thermodynamic (sorption) and barrier (diffusion) properties of A and B are uniform and constant in time, and not affected by the concentrations of substances s_A_ and s_B_. Hence, the one-dimensional distribution of $i=s_{A},s_{B}$ in and through the layers/components indexed from 1 to m reads along the spatial coordinate x at the time t:

(1) $$\frac{\partial C_{i,j}^{\left(t,x\right)}}{\partial t}=D_{i,j}\frac{\partial^{2}C_{i,j}^{\left(t,x\right)}}{\partial x^{2}}\;\mathrm{for}\;i=s_{A},s_{B};\;j=1\ldots m;\;L_{j-1}<x\leq L_{j}$$

where *C_i_*_,*j*_ is the local concentration of substance i in layer/component j of thickness $l_{j}$ and $D_{i,j}^{}$ its diffusion coefficient, with units in international system in kg·m^−3^ and m^2^·s^−1^, respectively; $L_{j}=L_{j-1}+l_{j}$ is the cumulated thickness with $L_{0}=0$.

Using the contact layer j=1 as a reference layer, the dimensionless version of Equation (1) becomes:

(2) $$\frac{\partial u_{i,j}^{\left(t,x^{*}\right)}}{\partial Fo}=\frac{D_{i,j}}{D_{i,1}}\frac{\partial^{2}u_{i,j}^{\left(t,x^{*}\right)}}{\partial x^{*}{}^{2}}\;\mathrm{for}\;i=s_{A},s_{B}\;\mathrm{and}\;j=1\ldots m$$

where $Fo=\frac{D_{i,1}}{l_{1}^{2}}t$, is the dimensionless time or Fourier number; $x^{*}=\frac{x}{l_{1}}$ is the dimensionless position; $u_{i,j}^{\left(t,x^{*}\right)}=\frac{L_{j}}{\sum_{k=1\ldots m}l_{k}C_{i,k}^{\left(t=0\right)}}C_{i,j}^{\left(t,x\right)}$ is the local concentration relative to the total amount in all layers/components. The most common assumption is to consider that the initial concentration in each compartment is uniform or zero and equal to $C_{i,j}^{\left(t=0\right)}$.

#### 2.3.4. Interface Conditions 

As concentration gradients are not “true” driving forces, Equation (2) does not apply at the interface between materials or with gas or liquid phases. Thermodynamical consistency is recovered by adding contact conditions which maintain the continuity of chemical potentials across each interface, denoted generically ∂Ω. This property is known as local thermodynamical equilibrium (LTE), and it entails not only a local chemical equilibrium for all constituents, including the polymer, but also a mechanical and thermal equilibrium on both sides of ∂Ω. By considering only the chemical potentials of migrants, the nature of sorption/desorption isotherms in each layer/component needs to be specified. Flory-type isotherms are the most relevant for thermoplastics and thermosets. They are suitable not only for homo and copolymers [23,24], but also for liquids simulating bioproducts such as methanol, ethanol, isopropanol, and water-ethanol mixtures [68,69,70]. At low concentration levels, Flory isotherms are linearized and recast into dimensionless Henry isotherms:

(3) $$q_{i,j}^{\left(t,x^{*}\right)}=\frac{L_{j}}{\sum_{k=1\ldots m}l_{k}C_{i,k}^{\left(t=0\right)}}p_{i,j}^{\left(t,x*\right)}=k_{i,j}\cdot u_{i,j}^{\left(t,x^{*}\right)}\;\mathrm{for}\;i=s_{A},s_{B}\;\mathrm{and}\;j=0\ldots m$$

where $q_{i,j}^{\left(t,x\right)}$ is the local dimensionless partial pressure of solute *i* at position *x* and time *t*. $k_{i,j}$ is the Henry-like coefficient, and it is related to the Flory-Huggins coefficient in the polymer layer j, $\chi_{i,j}$ as:

(4) $$k_{i,j}\left(T\right)\approx p_{i}^{sat}\left(T\right){\bar{V}}_{i}\exp\left(1+\chi_{i,j}\right)$$

with $p_{i}^{sat}\left(T\right)$ the saturation pressure of solute *i* at the absolute temperature *T* and ${\bar{V}}_{i}$ its molar volume. For any air layer, the ideal gas law leads to $k_{i,j}\left(T\right)=RT$ as used in Equation (11).

By denoting the position $x_{j,j+1}^{\partial \Omega }$ of the interface between layers/components *j* and j+1, LTE enforces:

(5) $$\frac{u_{i,j}^{\left(t,x^{*}=x_{j,j+1}^{\partial \Omega }\right)}}{u_{i,j+1}^{\left(t,x^{*}=x_{j,j+1}^{\partial \Omega }\right)}}=\frac{k_{i,j+1}}{k_{i,j}}\;\mathrm{for}\;i=s_{A},s_{B}\;\mathrm{and}\;j=1\ldots m-1$$

along with the continuity equation:

(6) $${\frac{\partial u_{i,j}^{\left(t,x^{*}\right)}}{\partial x^{*}}|}_{x^{*}=x_{j,j+1}^{\partial \Omega }}=\frac{D_{i,j+1}}{D_{i,j}}{\frac{\partial u_{i,j+1}^{\left(t,x^{*}\right)}}{\partial x^{*}}|}_{x^{*}=x_{j,j+1}^{\partial \Omega }}\;\mathrm{for}\;i=s_{A},s_{B}\;\mathrm{and}\;j=1\ldots m$$

#### 2.3.5. Boundary Conditions

Since the mass transfer rate is much faster in the liquids than in the polymers, the fluid or bioproduct F (*j* = 0) can be considered perfectly mixed, with a concentration $C_{i,0}^{\left(t\right)}$ (dimensionless form: $u_{i,0}^{\left(t\right)}$ except at the interface with the layer/component j=1, where a concentration gradient might exist due to a possible mass transfer resistance. The existence of the mass transfer boundary layer has been studied and interpreted in [64,71]. At the interface with F, the continuity between the diffusive flux in the solid and across the boundary layer leads to a different version of LTE at the Fluid-Packaging interface:

(7) $$-{\frac{\partial u_{i,1}^{\left(t,x^{*}\right)}}{\partial x^{*}}|}_{x^{*}=0}=\frac{h_{i}l_{1}}{D_{i,1}}\left(\frac{k_{i,1}}{k_{i,0}}u_{i,1}^{\left(t,x^{*}=0\right)}-u_{i,0}^{\left(t\right)}\right)=Bi_{i}\left(\frac{k_{i,1}}{k_{i,0}}u_{i,1}^{\left(t,x^{*}=0\right)}-u_{i,0}^{\left(t\right)}\right)$$

where $h_{i}$ is the mass transfer coefficient at the interface (on the fluid side) with SI units in m⋅s^−1^. $Bi_{i}=\frac{h_{i}l_{1}}{D_{i,1}}$ is the dimensionless equivalent mass Biot number. Equation (7) is known as a third kind or Robin boundary condition, as it involves both the concentration and its gradient. 

On the most external side, a conservative boundary condition is enforced to keep the distribution of migrants confined to the considered layers and components. For bags with bilayer composition, denoted AB| where the vertical bar “|” indicates the side intended to be in contact with F, the expected periodic configuration in stacks is AB|BAAB|BA or [AB|BA][AB|BA] if an overpackaging [] is used. Such configuration prevents any direct contact with contact between the contact surface | and A. It is adequately considered by implementing a symmetric or an impervious configuration:

(8) $$-{\frac{\partial u_{i,j}^{\left(t,x^{*}\right)}}{\partial x^{*}}|}_{x_{j}^{\partial \Omega }\;}=0$$

As a result, s_A_ can be present at the contact surface only after crossing the whole B thickness. Storing the original bilayer in rolls AB|AB| before sealing the bags triggers, on the contrary, a direct impregnation of the internal layer by direct contact. This phenomenon, coined “setoff”, is well documented and is responsible for instance, for the contamination of the internal side of packaging by external printed surfaces during storage in reels or stacks [68]. Such a condition is mathematically enforced by setting the periodic condition:

(9) $$p_{i,1}^{\left(t,x^{*}=0\right)}=p_{i,m}^{\left(t,x^{*}=x_{j}^{\partial \Omega }\right)}$$

#### 2.3.6. Macroscopic Mass Balance

Finally, the amount of substance i transferred to, or residual, in any layer *j* (including j=0) at the time t, denoted $m_{i,j}^{t}$, is obtained by integrating the solution over each layer/component. As the mass action holds, it is convenient to express transferred amounts relatively to their initial amounts. By neglecting the initial amount in F (j=0), one gets:

(10) $$\begin{array}{l} \frac{m_{i,j}^{t}}{\sum_{k=1\ldots m}m_{i,k}^{t=0}}=\frac{\int_{L_{j-1}/l_{1}}^{L_{j}/l_{1}}u_{i,j}^{\left(t,x^{*}\right)}dx^{*}}{\int_{0}^{L_{m}/l_{1}}u_{i,j}^{\left(t=0,x^{*}\right)}dx^{*}}\;\mathrm{when}\;j>0\\ \frac{m_{i,0}^{t}}{\sum_{k=1\ldots m}m_{i,k}^{t=0}}=1-\frac{\sum_{k=1\ldots m}m_{i,k}^{t}}{\sum_{k=1\ldots m}m_{i,k}^{t=0}}\;\mathrm{otherwise} \end{array}$$

#### 2.3.7. Superposition Principle for Many Components in the Same Assembly

The scope of Equation (10) can be extended by noticing that the contribution of each component to the overall amount is additive regardless of whether the systems are associated in series (multilayers) or parallel (bag with connected tubes). The associated theorem is stated as follows: If the contribution to cross mass-transfer (i.e., concentration kinetics, concentration profiles) of two (or more) components in an assembly is known, then the joint contribution of these two (or more) components in the same assembly is the sum of the two contributions (i.e., sum of two kinetics or concentration profiles). There is no limit or restriction to this theorem, while neither the number of materials in the assembly nor the nature of boundary conditions are modified. The mathematical demonstration relies on the linearity of the transport Equation (2), of interface conditions (5) and (6), and of boundary conditions (7)–(9) with concentration. The conditions of the commutativity of steps (i.e., independent of orders) are demonstrated in Reference [44] and reviewed extensively in Reference [31]. Commutativity is verified when the partition coefficients are kept constant (i.e., no effect of temperature, swelling). Worst-case scenarios can be constructed to remove sources of uncertainty (e.g., unknown partition coefficient, unknown layer contribution) by using superposition principles. Their construction is discussed in [31,46] and has been adopted in EU guidance for food contact materials [72]. 

#### 2.3.8. Similitude Models 

Case-studies S1 and S2, as well as their corresponding modeling with Equations (2)–(10), are exemplified for the dimensionless conditions listed in Table 2 and are discussed in the following sections. The dimensionless durations of step 1 (without contact with F), denoted $Fo_{step\;1}$ was arbitrarily chosen to 100 (i.e., two magnitude orders the diffusion time in the layer j=1). During the contacting step, the mass transfer resistance was assumed negligible in F, and a large mass Biot number (1000) was applied.

### 2.4. Cross-Mass Transfer with Direct Contact (Case-Study S1)

**Figure 1:**
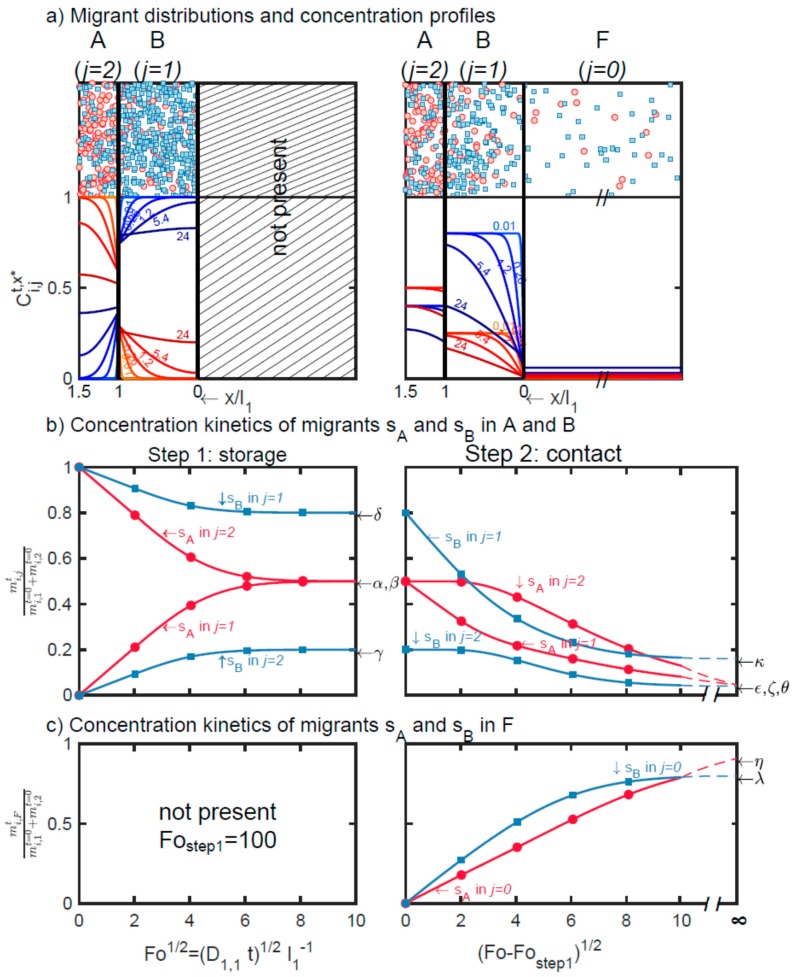
Cross-mass transfer of solutes s_A_ (●) and s_B_ (■) between A, B, and F (case-study S1): (**a**) Distribution of substances and corresponding concentration profiles at different dimensionless contact times *Fo* = 0.01, 0.2,…24; (**b**) dimensionless mass transfer kinetics of s_A_ and s_B_ in each layer (*j* = 1, 2); (**c**) dimensionless mass transfer of s_A_ and s_B_ in F (*j* = 0). Double indices {*i*,*j*} indicate the substance *i* = s_A_,s_B_ in layer *j* = 0,1,2. Greek letters refer to the corresponding equations in Table 3 to calculate the amounts at equilibrium: α (Equation (14) with *i* = 2); β (Equation (16) with *i* = 2); γ (Equation (14) with *i* = 1); δ (Equation (16) with *i* = 1); ε (Equation (15) with *i* = 2); ζ (Equation (17) with *i* = 2); κ (Equation (17) with *i* = 1); θ (Equation (15) with *i* = 1); η (Equation (18) with *i* = 2); λ (Equation (18) with *i* = 1).

Cross-mass transfer between A and B (step 1) and between A, B, and F (step 2) in case-study S1 are presented in Figure 1 as discrete particles displacing in two dimensions (Figure 1a) and one-dimensional concentration profiles (Figure 1b). The two representations are equivalent in the transverse direction. At interfaces, the distribution is discontinuous and fulfills both the mass conservation (Equations (6) and (7)) and the continuity of chemical potentials across them (Equation (5)). Figure 1b plots the corresponding one-dimensional continuous concentration profiles. The evolution of the concentrations averaged over each layer, with the contact time, is shown in Figure 1c.

The total contact time of step 1 is sufficiently long to equilibrate the net mass transfer between A and B. The substances s_A_ and s_B_ are distributed according to their apparent partition coefficients ${\left\{K_{i,\mathrm{reception}/\mathrm{source}}\right\}}_{i=1,2}=\frac{k_{1,1}}{k_{1,2}}=\frac{k_{2,2}}{k_{2,1}}$, chosen equal to 0.5. During step 2, the previous thermodynamical equilibrium is progressively displaced to F. Concentrations in F are diffusion-controlled and increase linearly with the square root of the duration of the contact. The maximum amounts transferable to F, are given in the general case by ${C_{0}|}_{t\to t_{eq}}=\sum_{j=0}^{n}\left(\frac{\rho_{j}}{\rho_{0}}\frac{l_{j}}{l_{0}}{C_{j}|}_{t=t_{0}}\right)/\left(1+\sum_{j=1}^{n}\left(\frac{k_{0}}{k_{j}}\frac{\rho_{j}}{\rho_{0}}\frac{l_{j}}{l_{0}}\right)\right)$ (Equation (2) in Reference [44]) with corresponding values summarized in Table 3. The depicted case-study demonstrates that similar migration levels can be achieved by substances s_A_ and s_B_ regardless of their initial distance to F.

### 2.5. Mass Transfer with Indirect and Intermittent Contact Between A and B (Case-Study S2)

Comparatively to S1, case-study S2 introduces several variations: An air layer separates A and B during step 1 (both materials can be easily separated); material A is discarded when B is in contact with F during step 2. As already discussed in Reference [17], adding an air layer does not prevent mass transfer but only slows down its rate. The driving force involves the solute volatility via its vapor saturation pressure, $p_{i}^{sat}\left(T\right)$ and its chemical affinity for the original condensed phase $\exp\left(1+\chi_{i,j}\right)$. Since the system AB remains closed during the first step, migration through the air layer (*j* = 2) is assumed to occur by diffusion and not by advection or thermal convection. Diffusion coefficients in the gas phase can be calculated directly from atomic structures with Fuller’s law [73].

**Figure 2:**
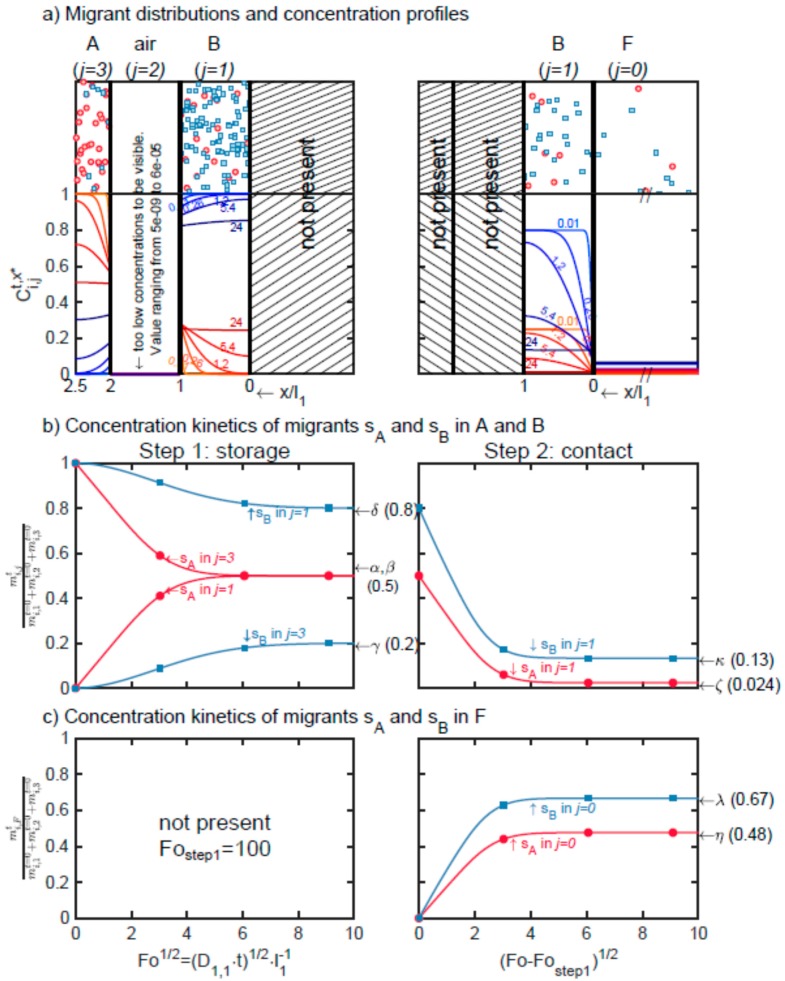
Cross-transfer of solutes s_A_ (●) and s_B_ (■) through the air layer (*j* = 2) separating A and B (case-study S2): (**a**) Distribution of substances and corresponding concentration profiles at different dimensionless contact times *Fo* = 0.01, 0.2,…24, (**b**) dimensionless mass transfer kinetics of s_A_ and s_B_ in each layer (*j* = 1, 2); (**c**) dimensionless mass transfer of s_A_ and s_B_ in F (*j* = 0). Double indices {*i*,*j}* indicate the substance *i* = s_A_,s_B_ in layer *j* = 0,1,2,3. Greek letters refer to the corresponding equations in Table 3 to calculate the amounts at equilibrium: α (Equation (19) with *i* = 2); β (Equation (20) with *i* = 2); γ (Equation (19) with *i* = 1); δ (Equation (20) with *i* = 1); ζ (Equation (21) with *i* = 2); κ (Equation (21) with *i* = 1); η (Equation (22) with *i* = 2); λ(Equation (22) with *i* = 1).

**STEP 1.** The results are plotted in Figure 2 with maximum rates of transfer summarized in Table 3. For the first step, Equation (3) in [17] provides an analytical solution on short times, highlighting its linearity with time and the main physicochemical parameters acting on the release of *i = s_A_* from A to B and, equivalently, of *i = s_B_* from B to A. Using notations of Equation (4), the rates of transfer of s_A_ or s_B_ on short contact times read:

(11) $$\begin{array}{l} \left(1-\frac{C_{i,j}^{t}}{C_{i,j}^{t=0}}\right)l_{i}\approx h_{i,2}\frac{k_{i,j}^{}}{RT}t=h_{i,2}\frac{p_{i}^{sat}\left(T\right){\bar{V}}_{i}}{RT}\exp\left(1+\chi_{i,j}\right)t\\ \;\mathrm{for}\;\left(i=s_{A},j=3\right)\;\mathrm{and}\;\left(i=s_{B},j=1\right) \end{array}$$

The size of the air compartment has a significant effect only for short-term contacts and for non-volatile substances (e.g., *s_B_*). 

**STEP 2**. During the second step of case-study S2 and because the layer A is removed, the total transferred amount of s_A_ is lower than in case-study S1, but it occurs faster. The amount of s_B_ is relatedly significantly higher in agreement with the larger size of B in the considered case.

### 2.6. Conditions Triggering Cross-Mass Transfer in Single-Use Systems

Mass diffusion in and through materials is a spontaneous physical process governed by the thermal agitation of atoms and molecules. The proximity of materials triggers cross-mass transfer and is particularly likely when the air is not renewed in the storage room. Removing the source does not prevent the subsequent potential risk of contamination of the contacting phase F (case-study S2). The amount transferred from source (A) to reception phase (B) during $t_{step\;1}$, denoted $m_{1,2}^{t_{step\;1}}$, can be envisioned as a random variate depending on the distribution of the duration of the contact between A and B (true contact or in the vicinity), denoted $f_{t_{step\;1}}\left(t\right)=pr\left(t_{step\;1}=t\right)$. Fundamental results or probabilistic modeling of migration are shown in References [31,67]. By denoting Ω(t), the monotonic loading model with $\Omega \left(t_{step1}\right)=m_{1,2}^{t_{step\;1}}$, and $\Omega^{-1}$ its inverse, the probability that $m_{1,2}^{\left(t_{step\;1}\right)}$ exceeds the value m is given by:

(12) $$Pr\left(m_{1,2}^{\left(t_{step\;1}\right)}\geq m\right)=1-\int_{0}^{m}f_{t_{step\;1}}\left(\Omega^{-1}\left(\mu \right)\right)\frac{d\Omega }{dt}\left(\Omega^{-1}\left(\mu \right)\right)d\mu \;\mathrm{for}\;\mathrm{any}\;m\geq 0$$

Equation (12) exemplifies the cumulative effect of exposure time on the contamination of B with substances originating from A. The quality of the contact affects the expression of L (see Equation (11)) and the rate of the cross-contamination, but similar amounts can be transferred on longer periods. 

For an independent observer, such as the end-user, as well, the contamination of F by substances s_A_ and s_B_ are equivalent and cannot be distinguished (observed symmetry in Equations (14), (16), (19), and (21)). In the perspective of the part-suppliers, the situations are different, as only B was intended to be in contact with F. As a rule of thumb and to encourage proper risk assessment, the following rules should be suggested:

The leaching potentiality of s_B_ (source material = B) to F, including its detection and quantification, should be primarily under the responsibility of the supplier of AB.

The leaching potentiality of s_A_ to F (source material = A) would be likewise under the responsibility of the manufacturer of AB or A. 

The end-user is responsible for the amounts of s_A_ and s_B_ transferred to F and on the occurrence of such transfer. 

Variations in transfer rates between case-studies S1 and S2 illustrate the large spectrum of possible cases. In S1, substances s_A_ and s_B_ are transferred to F in almost similar rates (see Figure 1c, step 2), whereas the thicknesses and initial amounts are very different (see Table 2). This scenario was constructed by considering that B was formulated to minimize the interactions with F; in consequence, potential migrants (s_B_ = monomers, additives) have a low chemical affinity for F. On the contrary, the part-supplier of A does not know the end-user application (contact of B with F). When the chemical affinity of s_A_ for F is higher than for s_B_, the final contamination of F by s_A_ is proportionally higher than for s_B_. Intermittent contacts (case-study S2) do not modify the figure. The maximum transferable amounts are proportional to the initial/residual concentrations in A and B and their thicknesses.

## 3. Results

Cross-mass transfer (so-called loading) from the overpackaging (part A) to the bag (part B) during storage (without liquid simulant) is firstly reported. The transfer from B to F (without the overpackaging) follows.

### 3.1. Cross-Mass Transfer from Overpackaging to Bags

#### 3.1.1. Distribution of Ԑ-Caprolactam in the Overpackaging Before Contact

The overpackaging (part A) was itself a multimaterial including a three-layer laminate including two tie layers. The internal layer in polyamide 6 (PA6) was sandwiched between two low-density polyethylene layers (LDPE) and constituted a significant source of Ԑ-caprolactam. High molecular weight PA6 is obtained, indeed, by the anionic ring-opening polymerization of Ԑ-caprolactam. The lactam ring is opened by a lactam anion, which is regenerated from the proton abstraction process induced by the primary amine anion from another monomer [74,75]. The linear polymerization of PA6 associated with this transacylation mechanism is, however, reversible and occurs in competition with the reverse reactions of cyclization. A thermodynamical equilibrium depending on polymerization conditions governs the residual concentrations of monomer, cyclic, and linear oligomers in the polydisperse linear polymer [76]. It is common to find residues up to several percent [77,78]. In our experiments, the content of extractible Ԑ-caprolactam in the overpackaging was of 1244 ± 280 mg⋅kg^−1^. This value corresponded to a residual content of 4850 ± 1090 mg⋅kg^−1^ in the sole PA6 before Ԑ-caprolactam distributes between all the layers of the overpackaging during its storage in reels at room temperature for several months. The partition coefficient of i = Ԑ-caprolactam between PA6 and LDPE was estimated from the following equation:

(13) $$\ln K_{i,\mathrm{PA}6/\mathrm{LDPE}}=\ln k_{i,\mathrm{LDPE}}-\ln k_{i,\mathrm{PA}6}=\chi_{i,LDPE}-\chi_{i,PA6}$$

The $\chi_{i,PA6}$ value of Ԑ-caprolactam in PA 6 was extrapolated to the storage (20 °C) and contact temperatures (40 °C) from Table 2 in Reference [79], −1.19 and −1.04, respectively. The value of $\chi_{i,LDPE}$ was estimated at ca. 0.76 according to the Flory–Huggins–Hansen model (see Equation 4.15 in [80]) combined with the group contribution method of Van-Krevelen (see chapter 7 in Reference [81]). The validity of this approach for polyolefins has been discussed by us previously in Reference [68]. Equation (13) yields $K_{\mathrm{PA}6/\mathrm{LDPE}}$ values of 7.03 and 6.05 at 20 °C and 40 °C, respectively. Since the total thickness of both LDPE layers represented 3.6 times the PA6 one, up to 37.5% of the amount Ԑ-caprolactam was assumed to be contained in LDPE layers before contacting the overpackaging with the bag. 

#### 3.1.2. Experimental and Simulated Loading Kinetics of Ԑ-Caprolactam in Bags

**Figure 3:**
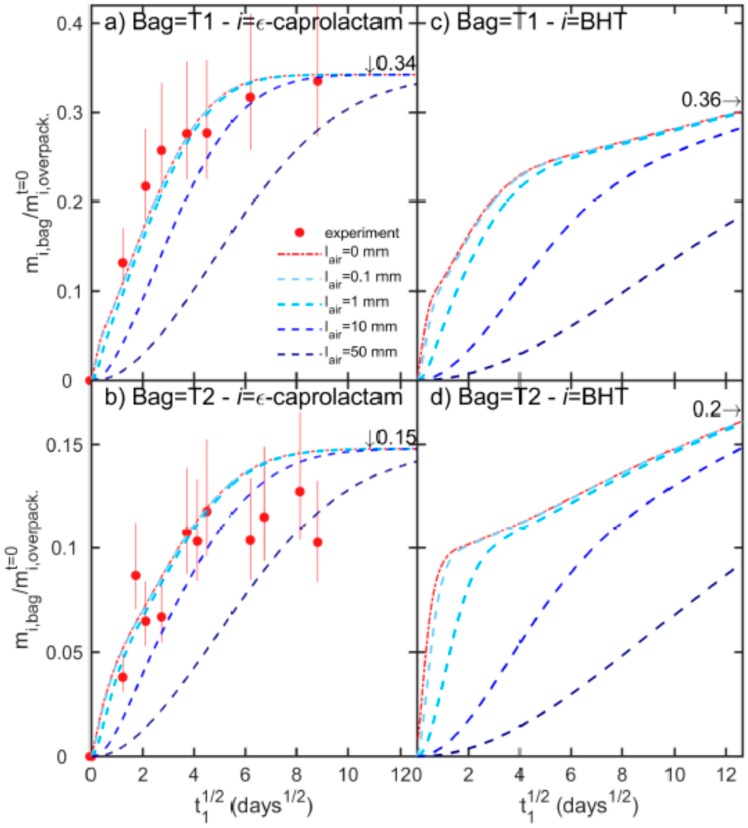
Loading kinetics (loading time *t_1_*) of Ԑ-caprolactam and Butylated hydroxytoluene (BHT) from overpackaging into multilayer bags T1 and T2 at 40 °C. Experimental and simulated kinetics are plotted as symbols and lines, respectively. $l_{air}$ represents the thickness of the gap filled with air between the overpackaging and the bag. Vertical bars show 95% confidence intervals.

The same type of overpackaging was put in contact with bags T1 and T2. The surface area of contact of the overpackaging was adjusted to fit the shape of each bag. Each system “one bag in one overpackaging” was isolated from its surroundings and other systems by placing it in a heat-sealed aluminum foil bag. The kinetics of mass transfer of Ԑ-caprolactam at 40 °C to the two bags are presented in Figure 3a,b for contact times up to three months. Results are normalized according to the initial amount of Ԑ-caprolactam in the overpackaging. Each experimental point corresponds to one single system, for which the mass balance was well verified after extraction between the bag, the overpackaging and the internal layer (also in LDPE) of the sealable external bag. A minimum recovery of 90 w% was used to accept experimental data. The maximum Ԑ-caprolactam amount transferred to the bag reached 34 w% and 15 w% in T1 and T2, respectively. 

Cross-mass transfer was analyzed by simulation with the following assumptions. The walls of each bag were modeled as trilayer materials including a central EVOH layer (see Table 6) and by neglecting tie layers. As EVOH is known to be partly a barrier to organic vapors with diffusion coefficients of dry EVOH 10 to 30 times lower than in LDPE [82,83,84,85], factor thirty was applied as an acceptable estimate. Contacts took place, indeed, in dry conditions as the relative humidity (RH) of the air in the bags ca. 50% RH at 20 °C decreased down to 16% at 40 °C. As verified experimentally, the polar materials (PA6, EVOH) dried slowly during the experiment. The values and assumptions to set the diffusion and partition coefficients of Ԑ-caprolactam are summarized in Table 7. The relative resistance to each layer ${\left\{\frac{k_{i,j}l_{j}}{D_{i,j}}\right\}}_{j=1..7}$ corresponded to 50, 46, 6,1, 7, 285, and 4 for T1 (from the most internal to the most external) and 47, 229, 22, 1, 7, 285, and 4 for T2 (idem). All resistances were relative to an air layer of 0.1 mm thick. 

Simulated cross-mass transfer between the overpackaging and the bags are shown in Figure 3a,b for a space filled with air between them varying from 0 mm (permanent and perfect contact) to 50 mm. The value of 50 mm was chosen as the upper bound of the spaces created by the imperfect packing of tubes, caps, and clamps attached to the bags. PA6 offered a resistance 285 times larger than an air gap of 0.1 mm, but the resistances were similar for a thickness of a 28.5 mm gap. In practice, air offered a significant resistance only above 10 mm, delaying cross-mass transfer but not the maximum amount of transferrable Ԑ-caprolactam. Equations (19)–(21) generalized to *j* = 1…7 layers predicted well maximum amounts at equilibrium, with theoretical values representing 0.342 and 0.148 the initial amount in the overpackaging, for T1 and T2 respectively. The higher sorption of T1 was associated with the higher chemical affinity of Ԑ-caprolactam for EVA than for LDPE. 

In practice, the contact between bags and overpackaging are imperfect with alternation of contact points and variable air separation. An average air thickness of 10 mm offers an additional delay to reach equilibrium varying from 36 days to 64 days. As the bags are usually vacuum packaged to maintain all components tightly and to avoid abrasion, air space is minimized and may be regarded as a slight retardant of migration. For prolonged storage, it is reasonable to assume that a very significant fraction of all volatile residues in the overpackaging will be transferred to single-use bags.

#### 3.1.3. Extrapolation of the Loading Kinetics for a Bulky Solute: BHT

Bulky solutes are expected to be less volatile and therefore not subjected to the same kinetics of release across an air gap (see the discussion in [17]). With a melting point at 70 °C, BHT is the prototype of substances with low volatility, which has been demonstrated to be transferable through the gas phase and to solid foods [86]. BHT is an antioxidant and antiaging commonly found in LDPE, with concentrations up to 500–1000 mg kg^−1^ [27]. In the overpackaging, BHT is consistently assumed to be distributed with more concentration in the LDPE layer than in the PA6 one. An LDPE-to-PA6 concentration ratio of 2.4:1 was applied. By comparison, it was of 1:6 for Ԑ-caprolactam.

The simulated loading kinetics of BHT are depicted in Figure 3c,d, and with the assumptions detailed in Table 8. Due to the preferential distribution of BHT in the LDPE layer directly exposed to the bag, the initial mass transfer rate was higher for BHT than for Ԑ-caprolactam. The higher sorption in bags T1 was associated with the higher chemical affinity of BHT for EVA than for LDPE. For both bags, a slowdown of migration occurred after four days once the depletion of the contact layer was enough advanced and not compensated by an equivalent flux from the opposite LDPE layer. The internal dry layer of PA 6 (glassy polymer) offered a mass transfer resistance of 10,000 times larger than the LDPE layer (rubbery polymer). Adding an air layer delayed cross-mass transfer so that the contributions of the PA 6 and opposite LDPE looked additive in time. As a result, the shoulder disappeared on the kinetics when the air thickness was increased. Figure 3c,d demonstrates the importance of contact conditions for low volatile compounds. A gap of 10 mm brings very similar delays between volatile and non-volatile compounds, up to ca. one month. One practical consequence is that imperfect and random contacts have the same effect as random contact times. The variability of storage conditions may affect significantly the capacity to extrapolate the experimental results from accelerated tests (short-time contact) to the entire product lifetime.

### 3.2. Sorption and Partitioning Properties with Liquid Simulants

In the presence of various materials and layers, mass transfer modeling is rapidly limited by the availability of partition coefficients or any similar concepts (activity coefficients, Henry coefficients, sorption isotherms) characterizing the relative chemical affinity. When each material or layer cannot be obtained independently, three strategies have been proposed to overcome previous limitations: i) Using effective partition coefficients to estimate maximum transferable amounts; ii) estimating the missing property by fitting a detailed model to experimental kinetics; or iii) changing the initial distribution in a way that cross-mass transfer can be overestimated. The last method proceeds by transferring the content of the unknown layer iteratively to the next one closer to the contacting phase until all missing partition coefficients have been removed. The details and justifications have been documented in the practical guide for food contact materials of the European Commission (see §4.2 of Reference [72]). The two first approaches were preferred in this study to reach both a global and detailed estimation of migration. The apparent partition coefficients between the entire bag and the simulant were experimentally determined from long-term experiments, whereas the specific barrier effect brought by the EVOH layer was fitted from migration kinetics. As discussed, hereafter, the combination of the two strategies was motivated by the necessity to reconstruct the distribution of each substance in the bag accurately before its migration in the liquid simulant occurs.

#### 3.2.1. Apparent Partitioning with Liquid Simulants

**Figure 4:**
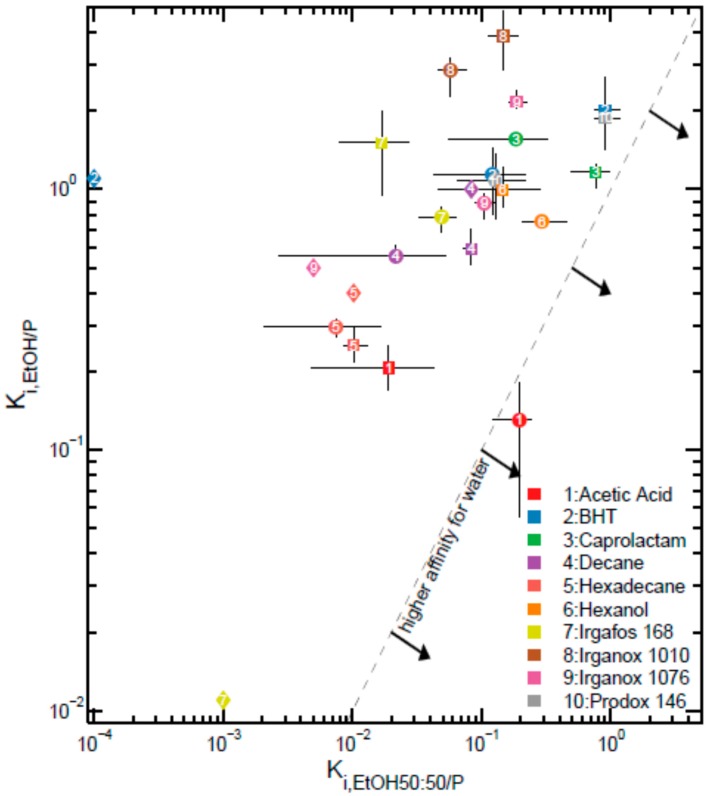
Comparison of the apparent partition coefficients, $K_{i,F/P}^{app}$, between F = ethanol and F = ethanol 50% for bags P = T1 (●) and P = T2 (■); 95% confidence intervals are indicated as horizontal and vertical lines. Values from Reference [69] for low-density polyethylene are also shown (♦).

The apparent partition coefficients between the liquid simulant (F) and the whole bag (P), denoted $K_{i,F/P}$, are compared in Figure 4 for *P* = T1 and T2 and *F* = absolute ethanol and ethanol 50%. All solutes have a higher chemical affinity for pure ethanol than for ethanol 50%, except acetic acid in wall bag T1. The values for pure LDPE (without EVOH) are also plotted and confirm the lower chemical affinity for EVOH comparatively to LDPE.

#### 3.2.2. Sorption Properties in Multilayer Structures

The effective Henry coefficient, $k_{i,eff}$, for a solute i within a structure including n layers or materials is given by $k_{i,eff}^{-1}=\sum_{j=1}^{n}\phi_{j}/k_{i,j}$, with ${\left\{\phi_{j}\right\}}_{j=1\ldots n}$ being the volume fraction of each layer or material. When only one $k_{i,j}$ value is unknown (e.g., $k_{i,EVOH}$), its value can be obtained directly from $k_{i,eff}$. When more than one value is missing (e.g., $k_{i,EVOH}$ and $k_{i,PE}$ in T2), additional information is required. We used in this study the full kinetics to identify the required Henry coefficients or equivalently their intrinsic partition coefficients with the contact phase $K_{i,F/j}=k_{i,j}/k_{F}$. Fitting experimental data with a mathematical model requires getting a good approximation of the initial distribution of the migrant between all layers. In the context of multiple steps, the distribution at the beginning of each step is affected by the history of mass transfer at the previous steps. The difficulty was circumvented by enabling equilibration between the “loading” and the “migration” step. As a result, the distribution of each migrant at the beginning of the migration step was updated iteratively to the guessed partition coefficients without changing the initial amount in the whole material.

**Figure 5:**
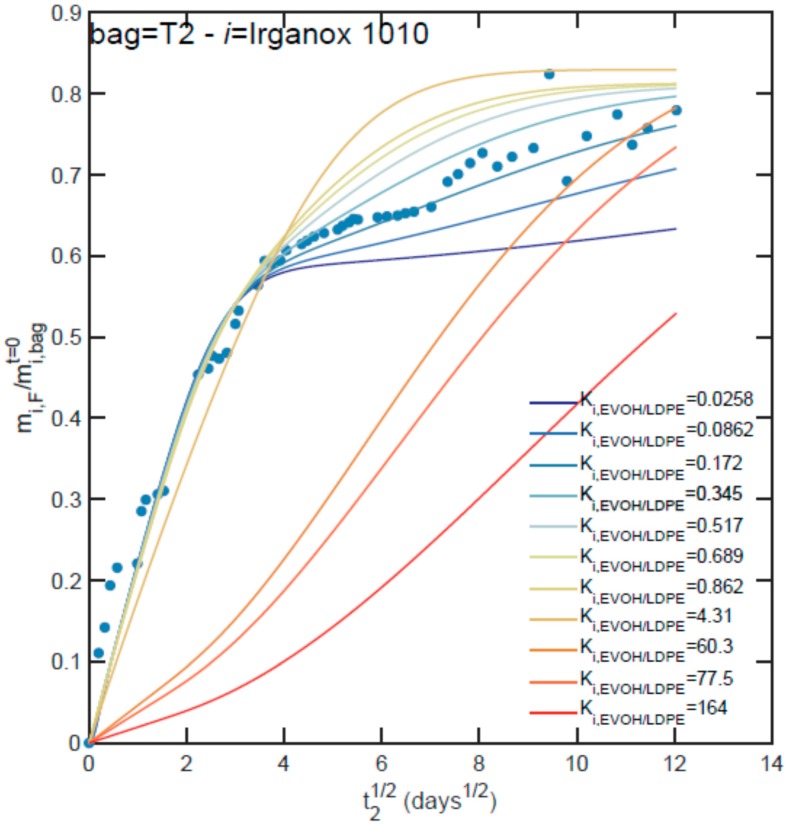
Example of the numerical identification procedure used to identify the unknown partition coefficient between EVOH and LDPE, *K_i,EVOH/LDPE_* = *k_i,LDPE_*/*k_i,EVOH_*, from migration kinetics (depicted case *i* = Irganox 1010 from bag T2).

The ability to identify missing partition coefficients from migration kinetics and apparent $K_{i,F/P}^{app}$ values is illustrated on the migration of Irganox 1010 from a loaded T2 bag in Figure 5. Due to the constraints introduced by the mass balance and the apparent partitioning, the quality of the fit of the kinetics is not monotonous with the ratio $K_{i,EVOH/PE}=k_{i,PE}/k_{i,EVOH}$. Indeed, the modifications of the initial distribution in the bag make it possible to detect unlikely ratios either on short-term contacts or on long-term contacts. In the presence of too low ratios, the contamination is brought almost exclusively by the layer in contact, whereas too large ones lead to delayed contamination dominated by the EVOH contribution. On intermediate values, two regimes can be identified successively: One in the square root of time corresponding to the transfer from the contact layer and one is linear with time corresponding to the contamination across the EVOH layer. Only values with ratios close to 0.2 reproduced the experimental kinetics. The likeliest values for each bag and substances are reported in Table 4 along with fitted diffusion coefficients when their values were not available in the literature. Arbitrarily and in agreement with Reference [82], diffusion coefficients in EVOH were considered thirty times lower than in the contact layer.

### 3.3. Desorption Kinetics of Model Solutes in Liquid Simulants

**Figure 6:**
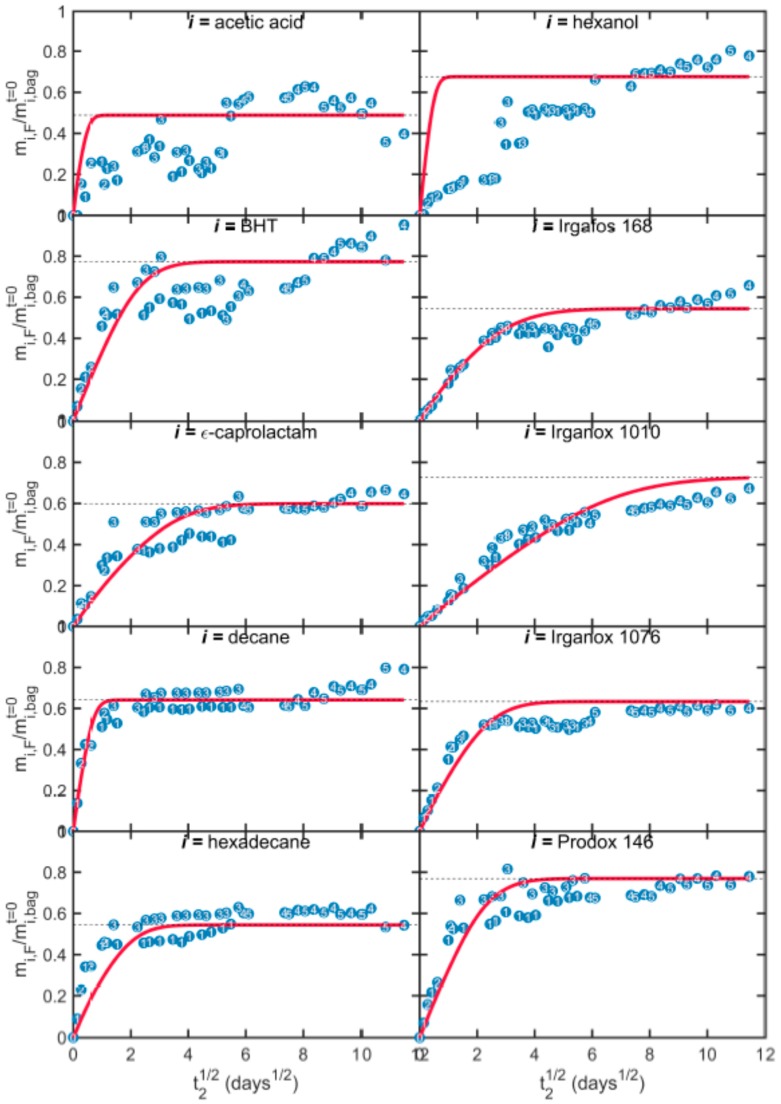
Experimental (symbols) and simulated (continuous lines) migration kinetics (migration time *t_2_*) from bags T1 to absolute ethanol at 25 °C. Depicted numbers indicate the index of the bag sampled. Horizontal dashed lines plot the theoretical equilibrium value.

**Figure 7:**
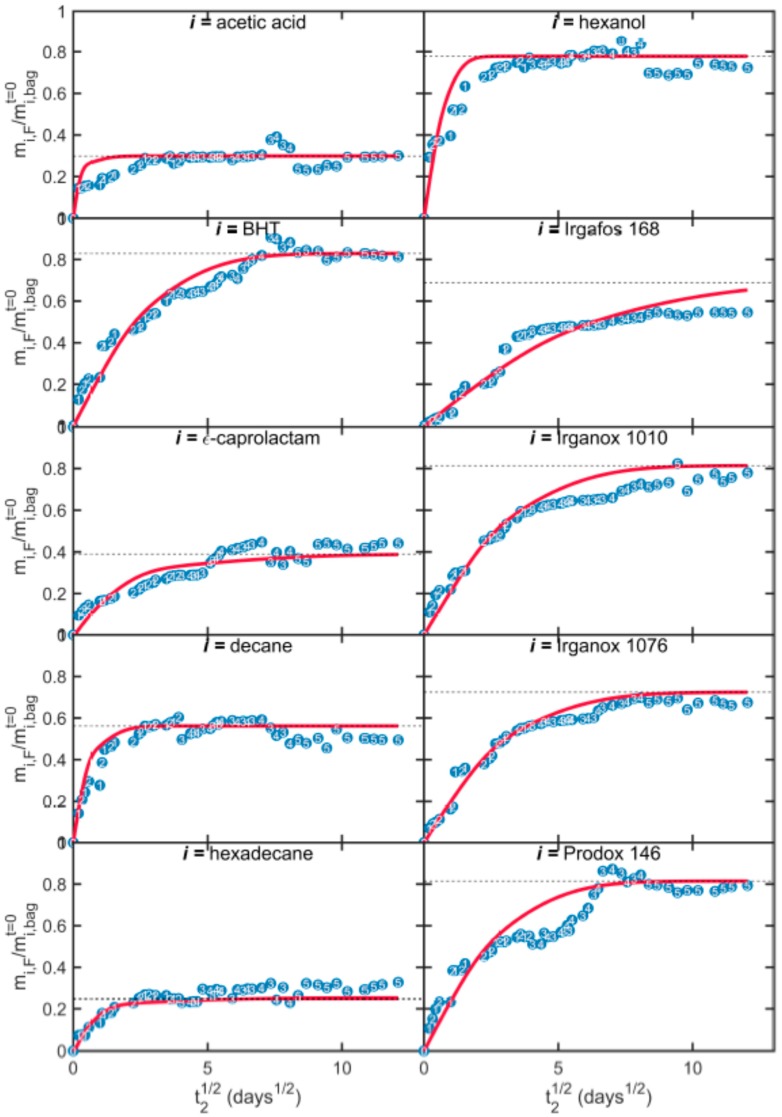
Experimental (symbols) and simulated (continuous lines) migration kinetics (migration time *t_2_*) from bags T2 to absolute ethanol at 25 °C. Depicted numbers indicate the index of the bag sampled. Horizontal dashed lines plot the theoretical equilibrium value.

The migration kinetics of the ten model solutes at 25 °C into ethanol are plotted in Figure 6 and Figure 7 for bags T1 and T2, respectively. Migration rates are normalized according to the amounts of solutes in the walls of bags T1 and T2. Experimental values were corrected from the amounts removed during sampling. Reconstructed kinetics are also depicted on the basis of numerical inputs detailed in Table 4.

Migration kinetics started without delay and were initially proportional to the square root of time. This first step was associated with the desorption from the contact layer (j=1) and was followed by a second step that was more linear in time and related to the contribution of the opposite layer (j=3). The transition from desorption to a permeation regime was associated with the barrier property of the EVOH layer, whose effective conductance is given by: $\frac{D_{i,EVOH}}{k_{i,EVOH}l_{EVOH}}$. Final migration rates varied between 40% to 80%. Both kinetics and maximum amounts were predicted satisfactorily by the models. The different behaviors of acetic acids and hexanol are associated with a progressive mass loss of volatile compounds from the opposite side of the bag (j=3).

### 3.4. Evaluation of the Risk of Cross-Mass Transfer

Previous desorption experiments and reconstructions were initiated with migrants in the bag initially at thermodynamical equilibrium. This worst-case assumption is only realistic for extended storage periods and severe transportation conditions. In most of the cases, single-use bags are used before the equilibrium between the overpackaging, the bag, and the air between them is reached. In practical cases, equivalent contact times $t_{1}$ between the bag and the overpackaging, as well as the storage temperature and the quality of contact, are not fully known. All these variations are equivalent to variations in contact times.

**Figure 8:**
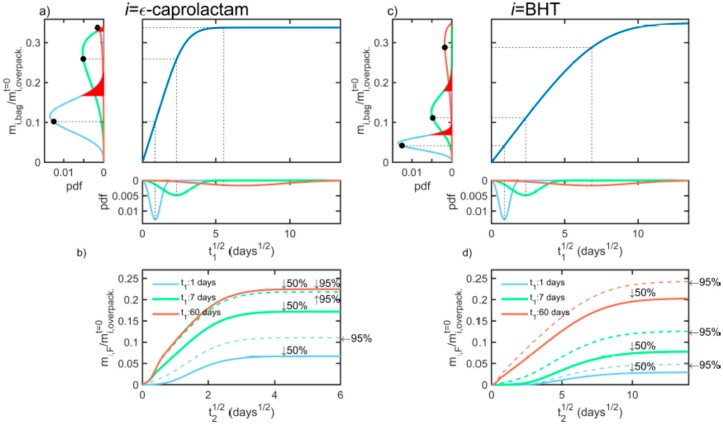
Probabilistic modeling of the cross-mass transfer of Ԑ-caprolactam (**a**,**b**) and BHT (**c**,**d**) from the overpack to the bag T1 (**a**,**c**) and finally to the liquid simulant F (**b**,**d**). The results are plotted for $t_{1}/{\bar{t}}_{1}$ distributed according to a Weibull distribution (scale parameter = 1; shape parameter = 3) and three values of ${\bar{t}}_{1}$ = 1, 7 and 60 days. The indicated percentiles correspond to the 50th and 95th value of the amount transferred in the bag T1 for each distribution.

Figure 8 illustrates the effect of the distribution of contact times $t_{1}$ (loading times) on the distribution of the cross-contamination of bags T1 by Ԑ-caprolactam and BHT and their subsequent risk of migration in F. The initial distribution in the overpackaging matched its equilibrium one (i.e., stored for several months before its use as packaging). According to Equation (12), the risk of cross-mass transfer can be evaluated either as the probability to exceed a given concentration value in F or as an upper percentile value (e.g., 95th) of the amount transferred to F after a contact time $t_{2}$ between T1 and F. Percentiles were preferred because they are similar to migration kinetics, as shown in Figure 8b,d. The computational procedure proceeds as follows. The cumulated distribution of the amount transferred is inverted to extract the value of $t_{1}$ matching the chosen percentile (50th or 95th). Finally, the concentration profile calculated at the corresponding $t_{1}$ value is used as an initial solution for the simulation of the migration step.

As suggested in References [31,67,87,88], contact times $t_{1}$ were decomposed, as the product of a scale parameter $\bar{t_{1}}$ and a unitary random variate $t_{1}^{*}$, following a Weibull distribution with a unitary scale and a shape parameter $s_{t_{1}}$. The results are plotted for $\bar{t_{1}}$ = 1, 7 and 60 days, and $s_{t_{1}}$ = 3. The differences between the 50th and the 95th percentiles of the contamination of *F* vanish for large $\bar{t_{1}}$ values but persist with $t_{2}$. Their differences represent the extra safety margin to be added to the deterministic case to account for the variability of contact times (or equivalent effects of temperature and quality of contact). For depicted cases, doubling the possible contamination in F would offer an acceptable choice for decision making. As the initial loading is proportional to the square root of $\bar{t_{1}}$, a safety factor of two would cover uncertainty in loading up to 4$\bar{t_{1}}$. Safety margins are partly recommended for low volatile substances and migration times much lower than loading times. The reasoning exemplified in Figure 8 can be complexified to handle variations in temperature and composition, as discussed in [31].

Besides the slower diffusion of BHT, the desorption behaviors of Ԑ-caprolactam and BHT differ from their different distributions in the bag after loading. For short loading times, BHT is mostly present in the external EVA layer (indexed j=3) delaying its desorption in F. This effect is minor with Ԑ-caprolactam. For long loading times, the migration rates of BHT and Ԑ-caprolactam are finally comparable due to their similar partition coefficients with ethanol (see Figure 4).

## 4. Materials and Methods

This study illustrates a realistic variant derived from the second case-study (see Table 1) for two types of bags (denoted T1 and T2). Ten substances representative of plastic materials were considered for s_A_. Specific attention was devoted to Ɛ-caprolactam, which is the residual monomer of polyamide 6 (PA6), the most frequent polymer in overpackaging complexes used in gamma-irradiated single-use systems. The scenario of cross-mass transfer is particularly complex to investigate and predict, as both the overpackaging and single-use bags are multilayers (six layers without counting air and F). The first step of cross-mass transfer, denoted “loading”, is the migration of Ɛ-caprolactam from the internal layer of the overpackaging to the layers of the bags in partial contact. The subsequent migration from the bags into a liquid contacting phase (F) was studied experimentally on bags spiked with ten surrogate migrants (Table 5), including Ɛ-caprolactam. Modeling and simulation were finally used to recover a full representation of the different cross-mass transfer.

### 4.1. Studied Materials and Configurations

**Figure 9:**
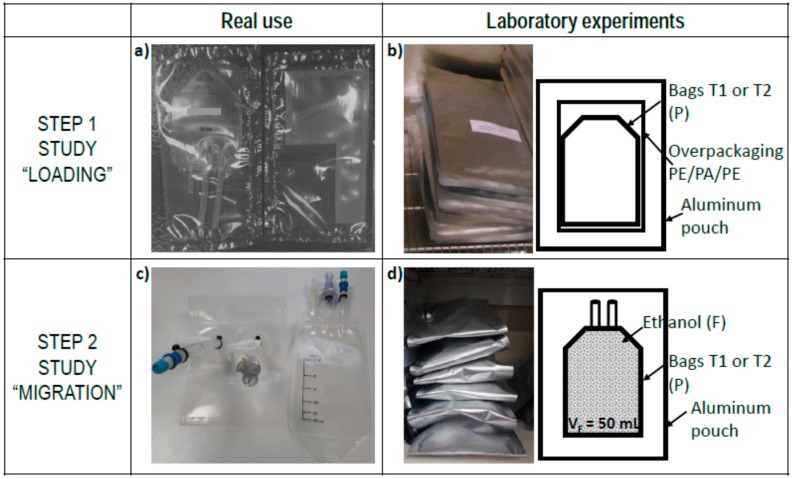
Experimental setup: (**a**) Real conditions of storage for multilayer bags T1 or T2 within their overpackaging; (**b**) experimental loading at 40 °C in sealed pouches and its schematic interpretation; (**c**) real conditions of use of bags T1 or T2 filled with a liquid (here ethanol); (**d**) experimental migration testing at 25 °C in sealed aluminum pouches and its interpretation.

The characteristics of the two single-use bags (denoted T1 and T2) and test configurations are summarized in Table 6. All bags were prepared by heat sealing together two trilayer laminates. Cross-mass transfer of Ɛ-caprolactam from the overpackaging (step 1 or “loading” study) was studied on bags T1 and T2 without any contacting phase (see Figure 9). The specific migration from the bag to the liquid in contact (step 2 or “migration” study) was assessed on similar but larger bags equipped with short tubes (3 and 2 for T1 and T2, respectively) to enable bag filling, sampling, and draining. It is important to note that all bag samples were randomized between the two studies, but that the bags were spiked with surrogates for the step 2 study.

### 4.2. Spiking Bags T1 and T2 for the Step 2 Study

Eight bags (either T1 or T2) involved in the step 2 study were formulated by immersing the bag in a 90:10 *v/v* ethanol-dichloromethane solution containing ten surrogate substances at a total concentration of ca. 2 g L^−1^ at 40 °C for seven days. The formulated bags were subsequently rinsed, dried, and equilibrated at 40 °C in aluminum foils for at least 15 days before being used to homogenize the distribution of surrogates in all parts of the bags. The final concentrations were estimated after equilibration and found uniform between bags. The overall concentrations for the bags and tubes are reported in Table 5, along with the main characteristics of the surrogate migrants. Surrogates were chosen to reproduce a broad range of chemical functions, polarity, and volatility.

### 4.3. Cross-Mass Transfer and Migration Testing

Migration conditions corresponding to step 1 and step 2 experiments are sketched in Figure 9.

Step 1 study (“loading”) was carried out by putting in contact the overpackaging with a neat bag, either T1 or T2. All systems were placed individually in sealed aluminum bags and stored in an oven at 40 °C for periods ranging from 1 to 100 days. For each sampling time, one system was removed, and both overpackaging and bags (either T1 or T2) were analyzed for concentration determination.

Step 2 study (“migration”) reproduced the solid–liquid contact by filling the spiked bags (either T1 or T2) with 50 mL of absolute ethanol as a liquid simulant. Bags were held horizontally in sealed aluminum bags in a climatic chamber at 25 °C for 1 to 120 days. During the experiments, permanent contacts between the liquid simulant and tubes were avoided. Migration kinetics were monitored by sampling regularly an aliquot of 0.5 mL among different bags, the number of samples did not exceed 10 per bag (total sampled volume 5 mL) to minimize the effect of volume on mass transfer. The bags were regularly weighed to consider any liquid mass loss due to sampling or leaks.

### 4.4. Determination of Initial Concentrations

Migrant concentrations were determined after three successive extractions from materials (overpackaging, T1, T2, and tubes). For the “loading” step (solid contact), 60 cm^2^ of film samples were cut into small pieces and extracted with 10 mL of pure dichloromethane. For the “migration” step (solid–liquid contact), 1 g of samples (bag and tube) were also cut into small pieces (approximate of 1 cm × 1 cm), placed in a glass vial and extracted with 10, 8, and 5 mL of the mixture of ethanol and dichloromethane 90:10 *v*/*v*. The whole successive extraction procedure lasted at least ten days at ambient temperature and involved an orbital stirrer with 200 rounds per minute. Transparent extracts were recovered after filtering through glass wool and a PTFE syringe with 0.45 μm of pore size (Sigma Aldrich). The distribution of surrogates in the different components of the single-use bag, including the wall bag and the connected tubes, is shown in Table 5. 

### 4.5. Migrant Concentration Analysis

Gas Chromatography (Agilent 6890N Network) associated with a Mass Spectrometer (Agilent 5975 Inert Mass Selective Detector) and Flame Ionization Detector with the separation column DB 5MS 29.8 m × 0.32 mm × 0.5 µm was used for concentration determinations. Helium was used as carrier gas at a flow rate of 1 mL∙min^−1^ and oven program was: Forty degrees celsius during 2 min; heating ramp of 5 °C∙min^−1^ up to 320 °C and hold for 20 min. The FID detector was operated at 320 °C. The mass spectrometer was operated at 220 °C; scans were carried out between 35 and 620 *m*/*z*.

For large solutes, liquid chromatography (Agilent) associated with a UV detector (Waters 2998 Photodiode Array) with the separation column Poroshell 120 EC-C18 2.7 µm, 4.6 mm × 100 mm was used. An isocratic program with a mixture of 5 % of tetrahydrofuran and 95 % of acetonitrile was applied for 20 min with a flow rate of 1 mL∙min^−1^ at 40 °C_._

### 4.6. Migration Modeling

Migration modeling was used to reconstruct, analyze, and extrapolate the kinetics of mass transfer across the different phases and layers during “loading” and “migration”. All simulations were performed with the open source project FMECAengine [91], enabling the combination of arbitrary configurations and steps.

#### 4.6.1. Cross-Mass Transfer from the Overpackaging to the Bag

Cross-mass transfer at 40 °C was simulated in one-dimension by considering an overpackaging on top of a single-use bag separated by gas layer with a thickness varying from 0 up to 50 mm. The system was symmetric and mimicked a stack of bags wrapped in their overpackaging. In agreement with experimental determinations, the loading of connected tubes and caps was neglected. Ԑ-caprolactam and BHT were taken as migration proxy from the packaging system and all parameters to simulate the cross-mass transfer of Ԑ-caprolactam and BHT are listed in Table 7 and Table 8, respectively. Initially, both substances were distributed in the overpackaging according to their relative chemical affinity for each layer. Corresponding Henry coefficients $k_{j}$ were calculated from $\chi_{j}$ values from the literature [69,79,80,81]. The diffusion coefficients in the air were inferred from Fuller’s law [73]. The diffusion coefficient of Ԑ-caprolactam in PA6 was extrapolated linearly from Equation (7) and Figure 8 in Reference [92]. The diffusion coefficients of small organic solutes were inferred from Tables 5 and 6 in [82] for EVOH and LDPE and Table 6 in Reference [22] for EVA. For BHT, diffusion coefficients in LDPE were experimentally assessed in Reference [30].

#### 4.6.2. Migration from Bags to F

Simulations of the desorption (migration) of ten surrogates at 25 °C were designed similarly. The initial concentrations were set from the experimental determinations during the step 2 study, with their detailed distributions between layers controlled by their relative chemical affinities. Desorption was simulated by assuming a one-sided contact (contact layer is indexed 1 and the contacting liquid 0) and an impervious boundary condition on the opposite side. The contributions of connected tubes and of the plastic parts outside the welding regions were neglected. These assumptions matched our experimental conditions: No contact of the tubes with F; clamps prevented mass transfer through the gas phase.

## 5. Conclusions

Cross-mass transfer between materials is not avoidable. It is strongly activated by temperature and occurs even in the presence of imperfect contacts or a gas layer separating them. These phenomena are sparingly discussed in the literature and appreciated mainly through experiments for biotechnological and pharmaceutical applications. The non-linearity of mass transfer with time in the presence of many materials and steps complicates their interpretation and our capability to extrapolate the results in the long term dramatically. Mathematical modeling becomes a necessary tool for both risk assessment and management. This study illustrates the principles of such detailed modeling on case-studies relevant for single-use applications. The mass transfer problem is broken into two steps: Loading and desorption. Despite the apparent complexity, detailed modeling in one-dimension enables to predict the final contamination for representative substances of plastics (monomers, oligomers, additives). The key parameters are the initial concentrations, the relative partition coefficients between materials and the contacting phase, and the diffusion coefficients. The latter is nowadays broadly available in the literature, and only partition coefficients remain scarce. In the context of multiple steps and materials, the initial distribution between the materials and chemical affinities are correlated unknowns. We devised a specific procedure to determine them simultaneously.

Simulation and mechanistic interpretation of mass transfer offer a robust framework to assess the potential cross-mass transfer. It is shown that adding an air layer delays mass transfer, but its effects are random with exposure-times and temperature. Reducing the initial concentration in source materials and surface areas is conversely much more beneficial. As an illustration for single-use applications, folding the bag in two will reduce by two the amount of packaging and in the same proportion the maximum amount of transferable substances. Additionally, multiplying twice the effective thickness of the folded bag will increase diffusion time by four during loading. During desorption, the surface area involved in the contamination is reduced twice as much (i.e., only one side of the bag is exposed) as the migration rate.

As already noticed in food contact applications and now for biopharmaceutical applications, assessing migration and cross-mass transfer require to consider not only the layers in contact but also all materials permanently present in the environment of the system of interest. Overpackaging and, possibly, cardboard used to stored and distribute single-use plastic systems are a significant source of migrants, which require proper risk management procedures, good manufacturing, and handling procedures. In aerated storage areas, there is a cut-off distance to which cross-mass transfer is likely (typically few centimeters) to occur. In closed storage areas or systems, the distance to consider could reach several meters.

## Figures and Tables

**Table 1:** List of case-studies.

CASE-STUDY	STEP 1: Storage Before Use		STEP 2: Contact During Use	
	Stacking	Equivalent Structure	Filled Bag	Equivalent Structure
S1 (e.g., single-use bag made from a laminate AB)	Laminate AB		Laminate AB in contact with F: AB-F	
	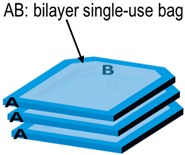	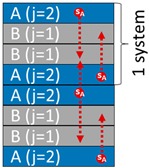	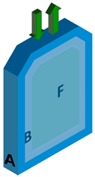	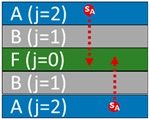
S2 (e.g., single-use bag made from a monomaterial B and wrapped in an over-packaging A)	Assembly A-air-B		B in contact with F: B-F	
	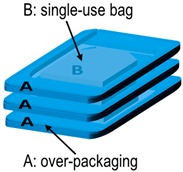	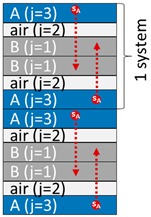	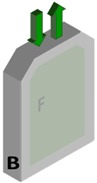	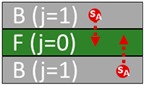

**Table 2:** Dimensionless parameters of case-studies S1 and S2 (see text for the details of notations).

CASE-STUDY	Parameters for Substance *i* = 1,2 (s_A_ = 2, s_B_ = 1) in Layer *j* = 0...m (F = 0, B = 1, A = 2 or 3)								
	Layer	$u_{1,j}^{t=0}$	$u_{2,j}^{t=0}$	$l_{j}/l_{1}$	$k_{1,j}/k_{1,1}$	$k_{2,j}/k_{2,1}$	$D_{1,j}/D_{1,1}$	$D_{2,j}/D_{2,1}$	$Fo_{step1}=D_{i,1}t_{step1}/l_{1}^{2}$
S1 (Figure 1)	A (*j* = 2)	0	1	0.5	2	0.5	0.5	0.5	100
	B (*j* = 1)	1	0	1	1	1	1	1	
	F (*j* = 0)	0	0	10	2	0.5	5000	5000	
S2 (Figure 2)	A (*j* = 3)	0	1	0.5	2	0.5	0.5	0.5	100
	air (*j* = 2)	0	0	1	10^8^	10^4^	10^8^	10^8^	
	B (*j* = 1)	1	0	1	1	1	1	1	
	F (*j* = 0)	0	0	10	2	0.5	5000	5000	

**Table 3:** Maximum amounts of s_A_ and s_B_ in the different layers (A,B,F) in case-studies S1 and S2.

		Step	Step 1: Storage	Step 2: Contact	
Case-Study					
			Structure **AB**	Structure **AB-F**	
**S1**	Layer A (*j* = 2)		The residual amounts of substances *i* = 1 (s_A_), 2 (s_B_) in A (layer *j* = 2) at the end of step 1 (at equilibrium) are: $m_{i,2}^{t_{step\;1}}=\frac{m_{i,1}^{t=0}+m_{i,2}^{t=0}}{1+\frac{l_{1}k_{i,2}}{l_{2}k_{i,1}}}$ (14)	The residual amounts of substances *i* = 1 (s_A_), 2 (s_B_) in A (*j* = 2) at equilibrium are: $m_{i,2}^{t\to \infty }=\frac{m_{i,1}^{t=0}+m_{i,2}^{t=0}}{1+\frac{l_{1}k_{i,2}}{l_{2}k_{i,1}}+\frac{l_{0}k_{i,2}}{l_{2}k_{i,0}}}$ (15)	
	Layer B (*j* = 1)		The complementary amounts in B (layer *j* = 1) are: $m_{i,1}^{t_{step\;1}}=\frac{m_{i,1}^{t=0}+m_{i,2}^{t=0}}{1+\frac{l_{2}k_{i,1}}{l_{1}k_{i,2}}}$ (16)	The residual amounts of substances *i* = s_A_,s_B_ in B (*j* = 1) at equilibrium are: $m_{i,1}^{t\to \infty }=\frac{m_{i,1}^{t=0}+m_{i,2}^{t=0}}{1+\frac{l_{2}k_{i,1}}{l_{1}k_{i,2}}+\frac{l_{0}k_{i,1}}{l_{1}k_{i,0}}}$ (17)	
	Layer F (*j* = 0)		As the layer F is not present, $m_{i,0}^{t_{step\;1}}=0$	The maximum amount of substances *i* = 1 (s_A_), 2 (s_B_) transferred to F (layer *j* = 0) reads: $m_{i,0}^{t\to \infty }=\frac{m_{i,1}^{t=0}+m_{i,2}^{t=0}}{1+\frac{l_{1}k_{i,0}}{l_{0}k_{i,1}}+\frac{l_{2}k_{i,0}}{l_{0}k_{i,2}}}$ (18)	
			Structure **A-air-B**	Structure **B-F**	
**S2**	Layer A (*j* = 3)		The residual amount of substances *i* = 1 (s_A_), 2 (s_B_) in A (layer *j* = 3) at the end of step 1 are: $m_{i,3}^{t_{step\;1}}=\frac{m_{i,1}^{t=0}+m_{i,2}^{t=0}+m_{i,3}^{t=0}}{1+\frac{l_{1}k_{i,3}}{l_{3}k_{i,1}}+\frac{l_{2}k_{i,3}}{l_{3}k_{i,2}}}$ (19)		
	
	Layer B (*j* = 1)		The complementary amounts in B (layer *j* = 1) are: $m_{i,1}^{t_{step\;1}}=\frac{m_{i,1}^{t=0}+m_{i,2}^{t=0}+m_{i,3}^{t=0}}{1+\frac{l_{3}k_{i,1}}{l_{1}k_{i,3}}+\frac{l_{2}k_{i,1}}{l_{1}k_{i,2}}}$ (20)	The residual amounts of substances *i* = 1,2 in B (*j* = 1) at equilibrium are: $m_{i,1}^{t\to \infty }=\frac{m_{i,1}^{t=0}+m_{i,2}^{t=0}+m_{i,3}^{t=0}}{\left(1+\frac{k_{i,1}}{l_{1}}\sum_{j=2,3}^{}\frac{l_{j}}{k_{i,j}}\right)}\left(1-\frac{1}{1+\frac{l_{1}k_{i,0}}{l_{0}k_{i,1}}}\right)$ (21)	
	Layer F (*j* = 0)		As the layer F is not present, $m_{i,0}^{t_{step\;1}}=0$	The maximum amount of substances *i* = 1 (s_A_), 2 (s_B_) transferred to F (layer *j* = 0) reads: $m_{i,0}^{t\to \infty }=\frac{m_{i,1}^{t=0}+m_{i,2}^{t=0}+m_{i,3}^{t=0}}{\left(1+\frac{k_{i,1}}{l_{1}}\sum_{j=2,3}^{}\frac{l_{j}}{k_{i,j}}\right)\left(1+\frac{l_{1}k_{i,0}}{l_{0}k_{i,1}}\right)}$ (22)	

**Table 4:** Numerical inputs for migration modeling of the step 2 study.

Solute *i*	BAG T1				BAG T2			
	$K_{i,F/EVA}$	$K_{i,F/EVOH}$	$D_{i,EVA}$ (×10^−14^) (m^2^∙s^−1^)	$D_{i,EVOH}$ (×10^−14^) (m^2^∙s^−1^)	$K_{i,F/PE}$	$K_{i,F/EVOH}$	$D_{i,PE}$ (×10^−14^) (m^2^∙s^−1^)	$D_{i,EVOH}$ (×10^−14^) (m^2^∙s^−1^)
**Acetic Acid**	0.16	0.23	147	6.97	0.09	0.23	209	6.97
**BHT**	0.57	0.97	2.52	0.18	1.12	0.97	5.39	0.18
**Ԑ-Caprolactam**	0.25	0.31	3.71	0.27	0.14	0.31	8.2	0.27
**Decane**	0.30	0.57	97.50	3.90	0.28	0.57	117	3.90
**Hexadecane**	0.20	0.26	10.10	0.44	0.07	0.26	13.3	0.44
**Hexanol**	0.35	0.59	187	4.07	0.82	0.59	122	4.07
**Irgafos 168**	0.20	0.38	4.11	0.06	0.52	0.38	5.78	0.06
**Irganox 1010**	0.45	0.94	1.21	0.24	0.50	0.94	2.13	0.24
**Irganox 1076**	0.29	0.57	7.70	0.22	0.60	0.57	6.73	0.22
**Prodox 146**	0.55	1.37	3.12	0.23	0.97	1.37	6.9	0.23

**Table 5:** Surrogate solutes used in step 2—experiments.

Chemical Name	Short Name/Brand Names	CAS Number ^a^	M (g∙mol^−1^) ^a^	$P_{i,sat}^{V}\;(\mathrm{Pa})$(25 °C)^b^	$C_{P}^{0}$(mg∙kg^−1^)	Structure
Acetic acid	Acetic Acid	64-19-7	60.05	2.14∙10^3^	c: 754 ± 63 d: 868 ± 56 e: 540 ± 15 f: 275 ± 16	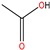
Hexan-1-ol	Hexanol	111-27-3	102.17	105	c: 533 ± 31 d: 699 ± 18 e: 213 ± 5.8 f: 213 ± 5.3	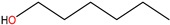
Azepan-2-one	Ԑ-caprolactam	105-60-2	113.16	0.483	c: 854 ± 61 d: 1033 ± 36 e: 630 ± 33 f: 463 ± 1.6	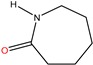
Decane	Decane	124-18-5	142.29	211	c: 2227 ± 33 d: 2471 ± 125 e: 1189 ± 75 f: 1333 ± 45	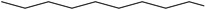
2,4-di-tert-butylphenol	Prodox 146	96-76-4	206.33	0.356	c: 1258 ± 29 d: 1097 ± 2.9 e: 324 ± 30 f: 573 ± 11	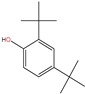
Butylated hydroxytoluene	BHT	128-37-0	220.35	0.236	c: 1159 ± 27 d: 1010 ± 2.7 e: 299 ± 28 f: 528 ± 10	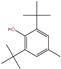
Hexadecane	Hexadecane	544-76-3	286.8	0.929	c: 4299 ± 129 d: 1660 ± 22 e: 2989 ± 123 f:1655 ± 65	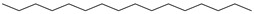
Octadecyl 3-(3,5-di-tert-butyl-4-hydroxyphenyl)propanoate	Irganox 1076	2082-79-3	530.87	4.51∙10^−11^	c: 2562 ± 58 d: 683 ± 27 e: 666 ± 13 f: 655 ± 77	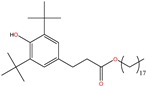
Tris(2,4-di-tert-butylphenyl)phosphite	Irgafos 168	31570-04-4	646.92	6.32∙10^−12^	c: 1907 ± 246 d: 417 ± 3.1 e: 172 ± 3.2 f: 795 ± 136	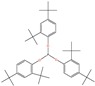
Pentaerythritol Tetrakis(3-(3,5-di-tert-butyl-4-hydroxyphenyl)propionate)	Irganox 1010	6683-19-8	1177.65	9.84∙10^−29^	c: 635 ± 37 d: 625 ± 9.1 e: 48 ± 6.9 f: 85 ± 0.9	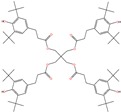

a: CAS number, molecular mass were from [89]; b: $\;P_{i,sat}^{V}$: estimated from EPI suite (application MPBPWI with Modified Grain Method), version 4.11, U.S. Environmental Protection Agency [90]; c: Initial concentration after equilibration of solutes in bag T1; d: Initial concentration after equilibration of solutes in tubes connected with bag T1; e: Initial concentration after equilibration of solutes in bag T2; f: Initial concentration after equilibration of solutes in tubes connected with bag T2.

**Table 6:** Summary of studied materials.

Material	Structure	Considered Experiment	Considered Migrant
Over-packaging	Trilayer laminate PE/PA6/PE (total thickness: 0.08 mm, with the relative thickness 2.3:1:1.3)	Step 1	Ԑ-caprolactam (monomer of PA6) with a residual concentration 1244 ± 280 mg∙kg^−1^ of laminate.
Bag T1	Trilayer laminate EVA/EVOH/EVA (total thickness *l* = 0.3 mm, with the relative thickness 53:1:6) Bag capacity: 50 mL Bag dimensions (L × W): 10.8 × 6.6 cm^2^	Step 1	Ԑ-caprolactam from overpackaging
	As the previous one. Bag capacity: 150 mL Bag dimensions (L × W): 15.5 × 9 cm^2^ Number of connected tubes: 3 (internal Ø = 2.5 mm, wall thickness = 0.5 mm, length = 20 mm)	Step 2	ten solutes (see Table 5) added by sorption
Bag T2	Trilayer laminate PE/EVOH/PE (total thickness *l* = 0.4 mm, with the relative thickness 10.2:1:4.8) Bag capacity: 50 mL Bag dimensions (L × W): 9.5 × 8 cm^2^	Step 1	Ԑ-caprolactam from overpackaging
	As the previous one. Bag capacity: 100 mL Bag dimensions (L × W): 13 × 11 cm^2^ Number of connected tubes: tubes (internal Ø = 3 mm, wall thickness = 0.7 mm, length = 100 mm)	Step 2	ten solutes (see Table 5) added by sorption

PE: polyethylene; PA6: polyamide 6; EVA: ethylene vinyl acetate; EVOH: ethylene vinyl alcohol; *l*: thickness; *L*: length; *W*: width; *r*: radius.

**Table 7:** Parameters for cross-mass transfer of Ԑ-caprolactam from the overpackaging to bags T1 and T2 at 40 °C.

**Bag T1**	Layer (index)	EVA (*j* = 1)	EVOH (*j* = 2)	EVA (*j* = 3)	air (*j* = 4)	PE (*j* = 5)	PA (*j* = 6)	PE (*j* = 7)
	$C_{j}^{t=0}$ (kg∙m^−3^)	0	0	0	0	0.58	3.62	0.58
	$k_{j}/RT$	1.83 × 10^−7^	3.83 × 10^−7^	1.83 × 10^−7^	1	2.47 × 10^−7^	3.96 × 10^−8^	2.47 × 10^−7^
	$D_{j}$ (m^2^∙s^−1^)	7.94 × 10^−14^	0.34 × 10^−14^	7.94 × 10^−14^	8.12 × 10^−6^	10.8 × 10^−14^	1.92 × 10^−16^	10.8 × 10^−14^
	$R=l_{j}k_{j}/D_{j}$	50	46	6	1	7	285	4
**Bag T2**	Layer (index)	PE (*j* = 1)	EVOH (*j* =2)	PE (*j* = 3)	air (*j* = 4)	PE (*j* = 5)	PA (*j* = 6)	PE (*j* = 7)
	$C_{j}^{t=0}$ (kg∙m^−3^)	0	0	0	0	0.58	3.62	0.58
	$k_{j}/RT$	2.47 × 10^−7^	3.83 × 10^−7^	2.47 × 10^−7^	1	2.47 × 10^−7^	3.96 × 10^−8^	2.47 × 10^−7^
	$D_{j}$ (m^2^∙s^−1^)	10.8 × 10^−14^	0.34 × 10^−14^	10.8 × 10^−14^	8.12 × 10^−6^	10.8 × 10^−14^	1.92 × 10^−16^	10.8 × 10^−14^
	$R=l_{j}k_{j}/D_{j}$	47	229	22	1	7	285	4

**Table 8:** Parameters for cross-mass transfer of BHT from the overpackaging to bags T1 and T2 at 40 °C.

**Bag T1**	Layer (index)	EVA (*j* = 1)	EVOH (*j* = 2)	EVA (*j* = 3)	air (*j* = 4)	PE (*j* = 5)	PA (*j* = 6)	PE (*j* = 7)
	$C_{j}^{t=0}$ (kg∙m^−3^)	0	0	0	0	4.0	2.1	4.0
	$k_{j}/RT$	2.37 × 10^−6^	2.90 × 10^−6^	2.37 × 10^−6^	1	1.63 × 10^−6^	3.20 × 10^−6^	1.63 × 10^−6^
	$D_{j}$ (m^2^∙s^−1^)	3.52 × 10^−14^	0.24 × 10^−14^	3.52 × 10^−14^	5.29 × 10^−6^	9.97 × 10^−14^	4.50 × 10^−17^	9.97 × 10^−14^
	$R=l_{j}k_{j}/D_{j}$	945	319	107	1	48	640,000	27
**Bag T2**	Layer (index)	PE (*j* = 1)	EVOH (*j* = 2)	PE (*j* = 3)	air (*j* = 4)	PE (*j* = 5)	PA (*j* = 6)	PE (*j* = 7)
	$C_{j}^{t=0}$ (kg∙m^−3^)	0	0	0	0	4.0	2.1	4.0
	$k_{j}/RT$	1.63 × 10^−6^	2.90 × 10^−6^	1.63 × 10^−6^	1	1.63 × 10^−6^	3.20 × 10^−6^	1.63 × 10^−6^
	$D_{j}$ (m^2^∙s^−1^)	9.97 × 10^−14^	0.33 × 10^−14^	9.97 × 10^−14^	8.12 × 10^−6^	9.97 × 10^−14^	4.50 × 10^−17^	9.97 × 10^−14^
	$R=l_{j}k_{j}/D_{j}$	316	1597	149	1	48	640,000	27
